# A dynamic spatiotemporal normalization model captures perceptual and neural effects of spatial and temporal context

**DOI:** 10.1371/journal.pbio.3003546

**Published:** 2025-12-01

**Authors:** Angus F. Chapman, Rachel N. Denison

**Affiliations:** Department of Psychological and Brain Sciences, Boston University, Boston, Massachusetts, United States of America; McGill University, CANADA

## Abstract

How does the visual system process dynamic inputs? Perception and neural activity are shaped by the spatial and temporal context of sensory input, which has been modeled by divisive normalization over space or time. However, theoretical work has largely treated normalization separately within these dimensions and has not explained how future stimuli can suppress past ones. Here, we introduce a dynamic spatiotemporal normalization model (DSTN) with a unified spatiotemporal receptive field structure that implements normalization across both space and time and ask whether this model captures the bidirectional effects of temporal context on neural responses and behavior. DSTN implements temporal normalization through excitatory and suppressive drives that depend on the recent history of stimulus input, controlled by separate temporal windows. We found that biphasic temporal receptive fields emerged from this normalization computation, consistent with empirical observations. The model also reproduced several neural response properties, including surround suppression, nonlinear response dynamics, subadditivity, response adaptation, and backwards masking. Further, spatiotemporal normalization captured bidirectional temporal suppression that depended on stimulus contrast, consistent with human behavior. Thus, DSTN captured a wide range of neural and behavioral effects, demonstrating that a unified spatiotemporal normalization computation could underlie dynamic stimulus processing and perception.

## Abstract 2

The effects of spatial and temporal context on sensory systems have mostly been studied independently of each other. This study shows that the modulation of visual perception and neural activity by these contextual domains can be captured by a dynamic spatiotemporal normalization model.

## Introduction

Although the world around us is highly dynamic, most theories and models of visual processing consider only a static snapshot of this constantly changing stream of visual stimulation. Less understood is the dynamic neural activity that supports visual perception, which exhibits several non-linear response properties that depend on time-varying stimulus input. Neurons in a range of cortical areas show sensitivity to time-varying stimuli [1], such as motion-sensitive neurons in V1 and MT, which can be characterized by their *spatiotemporal* receptive fields. More broadly, many neural responses and perception depend not just on current inputs but also on recent stimulus history [2–6], and can be modulated by future context as well [7,8]. These findings demonstrate that sensory systems are sensitive to temporal structure, which raises questions about what mechanisms exist to process dynamic inputs [9–11].

A powerful theoretical framework for visual processing is based on the principle of normalization, which has been proposed as a canonical neural computation [12]. Normalization is the idea that neurons can have suppressive effects on one another as a function of their tuning preferences, acting to “normalize” overall activity levels within a neural population [13]. Most normalization models are static, with the normalization computation operating across cortical space, which we refer to as spatial normalization. Temporal normalization, in contrast, is the idea that neural activity undergoes normalization across time [13]. Delayed normalization models implement temporal normalization by using a temporally filtered and delayed version of the excitatory input drive to compute the normalization signal [14,15]. Other models combine linear stimulus evoked responses with non-linear compressive—rather than divisive—computations to model how neurons respond to dynamic input [16,17]. All these models better predict neural response dynamics than linear-only models and capture several non-linear phenomena [15]. However, they also have important limitations. First, the entire excitatory time course must be known in advance, limiting their biological feasibility. Second, normalizing by a delayed copy of the excitatory drive means that only past stimuli can suppress future stimuli, not vice versa. Third, normalization is computed for each neural unit (neuron, voxel, electrode, etc.) in isolation, so to date these models have not considered how temporal normalization may operate across a broader suppressive pool, which has been critical for the success of spatial normalization models.

Here, we introduce the dynamic spatiotemporal normalization model (DSTN), a model of visual processing in which spatial and temporal normalization are integrated within a unified receptive field-based spatiotemporal normalization computation. Model neurons are situated in a neural network architecture, performing real-time processing of continuous visual inputs. We analyzed the response properties of this model, focusing on its time-varying behavior and modulation by temporal context. By unifying normalization across space and time, we find that DSTN captures key non-linear temporal response properties of neurons and predicts human behavior. Critically, unlike previous dynamic normalization models, it can produce bidirectional temporal suppression between stimuli in a sequence, allowing for empirically demonstrated effects of temporal context that operate both forward and backward in time. The current work builds on the normalization model of dynamic attention developed by Denison, Carrasco, and Heeger [18], which generalized the normalization model of attention [19] to the time domain, though for simplicity we do not include attention in the current model. DSTN advances previous work by implementing excitatory and suppressive drives that depend on recent stimulus history, imbuing the model neurons with receptive fields and normalization computations that are inherently spatiotemporal.

## Results

### The dynamic spatiotemporal normalization model (DSTN)

**Fig 1:**
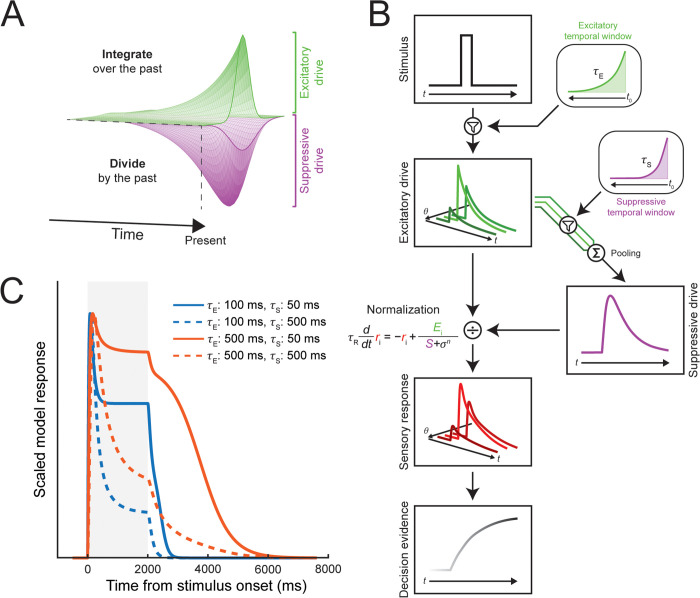
Architecture of the Dynamic Spatiotemporal Normalization model (DSTN). **A)** Spatiotemporal receptive field structure. Model sensory neurons integrate over the recent history of stimulus input, while contributing to a suppressive pool that also extends into the past. Model neurons can be tuned to different spatiotemporal properties of the stimulus input as well as to other stimulus features. **B)** Schematic of computations in DSTN. The model receives time-varying stimulus input (here, an oriented stimulus with a specific contrast), which is continuously filtered by the excitatory temporal window. The stimulus input produces excitatory drives in model neurons tuned to different orientations (*θ*). The excitatory drives of each neuron are continuously filtered and pooled to calculate the suppressive drive, which in turn normalizes the excitatory drives to compute sensory responses. Sensory layer activity is fed into the decision layer, which accumulates evidence about the stimulus orientation. **C)** Example sensory layer responses to a stimulus presentation (shaded gray region) as a function of different excitatory and suppressive temporal window parameters.

We introduce a model of dynamic visual perception building on [18] that produces neural responses and behavioral outputs. The model simulates sensory responses to stimulus input through neurons that are tuned to specific spatial locations and feature values and, critically, integrate inputs over the recent past, giving them spatiotemporal receptive fields (Fig 1A). As in previous models, spatial and feature dimensions are treated equivalently [19], with normalization across these dimensions determined by the tuning properties of model neurons. The sensory responses are read out by a decision layer, which accumulates evidence toward a particular behavioral output. Importantly, we include spatiotemporal normalization at each stage of processing, with excitatory and suppressive drives that contribute to the final neural response (Fig 1B). Responses are continuously updated at each time point with differential equations, allowing us to examine how model parameters affect the dynamics of neural responses as well as the final behavioral output. Thus, the model generates neural responses continuously at each time step (Fig 1C), at a level of abstraction that allows us to focus on how excitatory and suppressive components affect population activity separate from single unit physiology or neural circuit dynamics.

The core of the model is the spatiotemporal normalization computation, which divides the spatiotemporal excitatory drive by the spatiotemporal suppressive drive. To investigate the effects of temporal normalization, we implemented temporal receptive fields in model sensory neurons by allowing the excitatory drive to depend on stimulus input at previous points in time. Specifically, at each time point, the stimulus time course was weighted by an exponential decay function, the excitatory temporal window, such that input at more distant points in the past had less effect on the neuron’s response. The time constant (*τ*_E_) determined the relative weight given to stimuli at each point in time. When *τ*_E_ is zero, the model neuron only receives excitatory drive when stimulus input is present, but increasing *τ*_E_ results in responses that are driven even after the stimulus ends. To generate the neuron’s suppressive drive, its excitatory drive was further weighted by an exponential decay function, the suppressive temporal window, with a separate time constant (*τ*_S_). Suppressive drives were then pooled across all neural units to produce the suppressive drive of the neural population, which was used to normalize the response of each individual neuron, as in previous work [18,19]. The suppressive drive is thus dependent on both the excitatory and suppressive time constants, with the effect that suppression always acts later and across longer time intervals than excitation (even when *τ*_S_ < *τ*_E_). We performed a series of simulations varying aspects of the stimulus input to sensory layers to examine whether the model could reproduce several different non-linear response properties and to determine the effect of the excitatory and suppressive time constants on these response dynamics. Time constants varied across simulations, but in each simulation, all model neurons in the population had the same time constants.

### Stimulus history differentially affects model drives and responses

**Fig 2:**
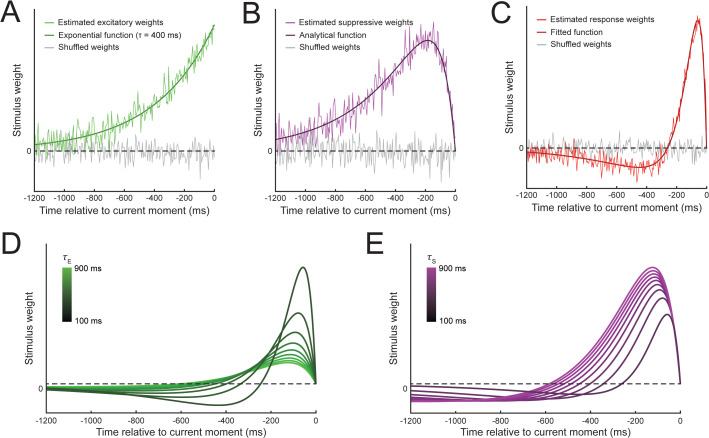
Reverse correlation analysis of model drives and sensory response reveals biphasic temporal receptive field. Stimulus weights were estimated for **A)** excitatory drives, **B)** suppressive drives, and **C)** sensory layer responses. Examples shown reflect simulations with *τ*_E _= 400 ms and *τ*_S _= 100 ms. Each set of estimated weights were fitted using different functional forms, (see main text). We also estimated the sensory layer weight functions (i.e., temporal receptive fields) across a range of parameters for **D)** excitatory temporal windows, and **E)** suppressive temporal windows. While varying one parameter, the other was fixed at an intermediate level (400 ms) for visualization purposes; the pattern of results did not depend on the specific fixed value.

The dependence of a neuron’s current response on recent stimulus inputs defines its temporal receptive field. We used reverse correlation to determine how excitatory and suppressive temporal windows interact to shape the functional temporal receptive fields of model neurons. We first examined how the model responses depended on stimulus history for a specific set of excitatory and suppressive temporal windows (*τ*_E _= 400 ms, *τ*_S _= 100 ms). Random stimulus input was fed into the model sensory layer, driving variable activity across simulations. We calculated how the presence of stimuli up to 1,200 ms in the past affected model neuron responses at the current moment by correlating the random stimulus vectors with the observed excitatory drive, suppressive drive, and sensory layer response. As expected, the excitatory drive depended most strongly on input close to the current time, with weights back in time following the exponential excitatory temporal window (Fig 2A). The suppressive drive depended on times in the recent past, and could be approximated by a convolution of the excitatory and suppressive temporal windows (Fig 2B), consistent with how suppressive drives are a temporally weighted sum of all model neurons’ excitatory drives, meaning both *τ*_E_ and *τ*_S_ affect the shape of the suppressive drive.

Reverse correlation revealed that this implementation of normalization generates a biphasic temporal receptive field (Fig 2C), where stimulus input close to the current time drives model neuron responses and input further in the past reduces responses. Notably, the biphasic stimulus weighting function was not built directly into the model, but emerged through the interaction between the excitatory and suppressive temporal windows. A similar biphasic function in time has been found in empirical recordings from visual neurons [20–22], and is similar to those used in models of neural temporal dynamics [15–17]. The estimated response weights were well fitted by a difference of Gamma functions, following previous work [15,20].

We performed additional simulations to explore the effect of the excitatory and suppressive time constants on the shape of the temporal receptive fields. Increasing the excitatory time constant, while holding the suppressive time constant fixed (*τ*_S _= 400 ms), resulted in a scaling and extension of the temporal receptive field to times further in the past (Fig 2D), as stimuli at these times fell into the longer excitatory temporal windows—a “flattening out” of the response profile. Increasing the suppressive time constant, with the excitatory time constant fixed (*τ*_E _= 400 ms), also resulted in an extension of the temporal receptive field to past times (Fig 2E), although this was a consequence of the suppression being distributed over a longer time span, resulting in less suppression for more recent times. Increasing the suppressive time constant also resulted in longer periods of suppression that extended further into the past, as would be expected given the longer suppressive windows. Thus, the excitatory and suppressive temporal windows generated temporal receptive fields that exhibit a variety of response profiles depending on each time constant.

### Reproducing signatures of spatial normalization

**Fig 3:**
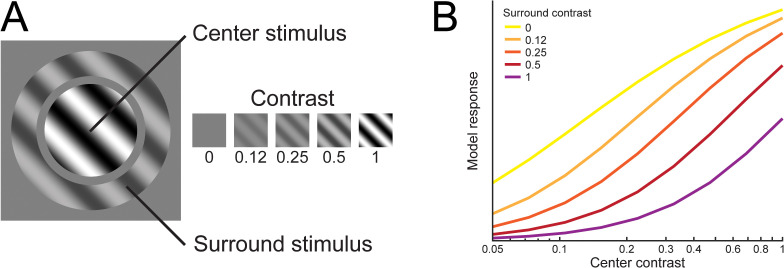
Model sensory neurons exhibit contrast-dependent surround suppression, a signature of spatial normalization. **A)** We measured the response of neurons tuned to the orientation and spatial location of the center stimulus while manipulating the contrast of both the center and surround stimuli (in this example, center contrast = 1, while surround contrast = 0.5). The center and surround were presented simultaneously to model neurons for 100 ms, and model temporal window parameters were fixed across simulations (*τ*_E _= 400 ms, *τ*_S _= 100 ms). **B)** Responses showed the expected contrast response function as the center stimulus contrast increased, as well as increasing suppression from the surround as its contrast increased.

Before turning to the time-dependent aspects of the model, we sought to ensure that DSTN could capture effects associated with static spatial normalization as expected. One finding commonly attributed to normalization is surround suppression: the suppression of a neuron’s response when a competing stimulus is placed in the region surrounding its excitatory receptive field (Fig 3A) [23,24]. The explanation given by spatial normalization is that while the neuron may not be responsive to stimuli placed in the surround region alone, other neurons that are tuned to stimuli in those spatial locations contribute to the normalization pool, such that activity driven by surrounding stimuli suppresses neurons tuned to center stimuli. We reproduced this effect in DSTN by varying the contrast of the center and surround stimuli independently [12,25]. The model responses exhibited sigmoidal contrast response functions, as is typical of normalization models [13,26]. While the model response to the center stimulus increased monotonically as a function of contrast, its response was simultaneously suppressed by a stimulus in the surround, with stronger suppression for higher surround contrasts (Fig 3B). Thus, DSTN captures a key signature of spatial normalization.

### Reproducing known temporal properties of neuronal responses

We next asked whether DSTN could reproduce several known non-linear temporal response properties of neurons: 1) transient-sustained dynamics, 2) subadditivity, and 3) response adaptation. For each property we asked whether excitatory temporal windows, suppressive temporal windows, or both were necessary to reproduce the property and how the non-linear temporal effects depended on the excitatory and suppressive time constants in the model.

#### Transient-sustained dynamics.

**Fig 4:**
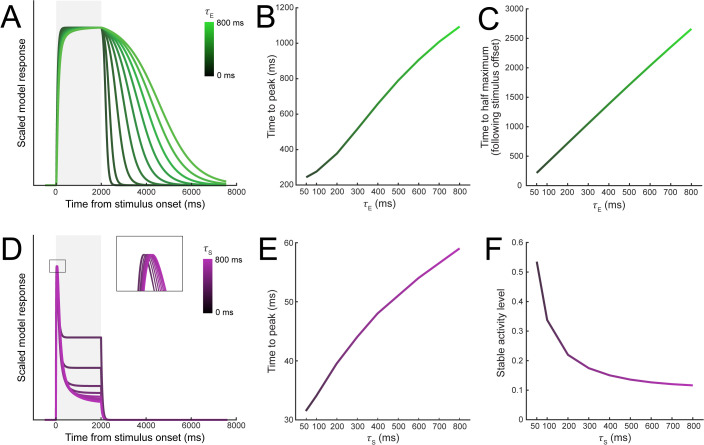
Excitatory and suppressive time constants differentially contribute to transient-sustained response dynamics. **A)** Effect of the excitatory time constant, ranging from 0 to 800 ms in 100 ms steps. Shaded region shows the stimulus presentation period. **B)** Time to peak for sensory responses as a function of *τ*_E_. **C)** Time for sensory responses to reduce to 50% of maximum as a function of *τ*_E_. **D)** Effect of the suppressive time constant on model sensory responses. The insert shows the early peak responses. **E)** Time to peak for the sensory responses as a function of *τ*_S_. **F)** Stable activity level of sensory responses, relative to the peaks, reached by the end of the stimulus presentation period as a function of *τ*_S_.

Neurons typically show an initial transient response followed by sustained activity to a prolonged stimulus [27,28]. We found that the suppressive temporal window was necessary to reproduce these dynamics. When varying the excitatory time constant with no suppressive temporal window (*τ*_S _= 0), responses increased gradually towards a stable activity level following stimulus onset and decreased only after stimulus offset, consistent with linear predictions [15]. Higher *τ*_E_ resulted in slower rise times (Fig 4A) and consequently longer times to peak (Fig 4B), as well as more prolonged responses after stimulus offset (Fig 4C). The model exhibited slower response dynamics with increasing *τ*_E_, because longer integration windows provided excitatory drive from stimulus input further in the past. In contrast, when varying the suppressive time constant alone (*τ*_E _= 0), model responses showed a more typical transient response, with an initial peak shortly after stimulus onset that decreased to a stable level before stimulus offset (Fig 4D). Higher *τ*_S_ resulted in longer times to peak (Fig 4E) and a lower stable activity level relative to peaks (Fig 4F), because suppression integrated more slowly but reached greater levels overall. Varying both time constants generated model responses with a diverse range of temporal profiles (Fig 1C). Thus, suppression alone, or a combination of excitatory and suppressive temporal windows, can capture typical neural dynamics to prolonged stimulus presentations.

#### Subadditivity.

**Fig 5:**
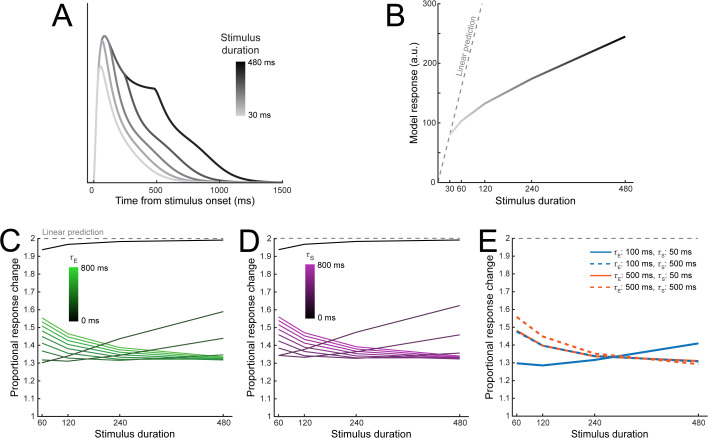
Subadditivity of model responses is robust across excitatory and suppressive time constants. **A)** Sensory layer responses over time in response to stimuli of varying durations (30, 60, 120, 240, or 480 ms), using *τ*_E _= 100 ms and *τ*_S _= 50 ms. **B)** Model responses calculated as the area under the curve of the sensory responses across the full simulated trial. Linear predictions are calculated relative to the model response for the 30 ms stimulus duration. **C)** Effect of *τ*_E_ and D) *τ*_S_ on subadditivity as a function of stimulus duration. Subadditivity was measured by taking the model response for each stimulus duration and dividing it by the response for a stimulus presented for half of that duration. Values below 2 represent subadditive responses. **E)** Proportional response changes as a function of different excitatory and suppressive time constants. The solid blue line corresponds to the time constants used in panel **A.**

Another hallmark of non-linear response dynamics is that neural responses display temporal subadditivity: doubling the duration of the stimulus leads to a less than doubling of the response amplitude [14,29]. Increasing the stimulus duration in DSTN resulted in more prolonged model responses (Fig 5A) that were strongly subbadditive, as quantified by the area under each curve (Fig 5B). We found that increasing either *τ*_E_ or *τ*_S_ was sufficient to produce subadditive model responses (Fig 5C and 5D); when both time constants were zero, model responses fell just below the linear prediction, showing only slight subadditivity due to the time constant inherent to the model’s recursive computation (*τ*_R_, equation (6)). For values of *τ*_E_ and *τ*_S_ above zero, each doubling of the stimulus duration increased responses by only ~1.3–1.6×. Combining different parameter values produced similar levels of subadditivity (Fig 5E), where lower values of each time constant resulted in relatively more subadditivity at short stimulus durations, with higher values resulting in more subadditivity at longer stimulus durations.

#### Response adaptation.

**Fig 6:**
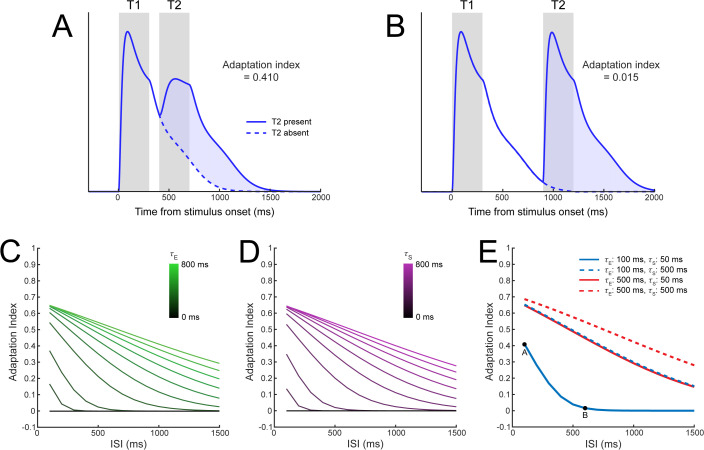
Response adaptation arises from either excitatory or suppressive temporal windows. **A)** Model sensory responses as a function of the presence of a preceding stimulus with an interstimulus interval of 100 ms, using *τ*_E _= 100 ms and *τ*_S _= 50 ms. Response adaptation is demonstrated by the reduced response to T2 (blue shaded region) relative to T1. **B)** Response adaptation was nearly eliminated when the ISI was increased to 600 ms. **C)** Effect of *τ*_E_ and D) *τ*_S_ on response adaptation for different ISIs. An adaptation index of zero represents no change in responses; positive indices show response adaptation. **E)** Combining different values of the *τ*_E_ and *τ*_S_ produces a range of response adaptation profiles. Parameters corresponding to the simulations in panels A and B are indicated by labeled dots on the solid blue line.

Neurons exhibit response adaptation, such that responses are lower following repeated stimulus presentations [28,30–33]. The magnitude of this adaptation depends on the interstimulus interval (ISI), with shorter ISIs resulting in stronger response adaptation. We observed a similar pattern in model simulations, where responses to the second stimulus (T2) in a sequence of two identical stimuli were lower when the ISI was shorter (e.g., 100 ms; Fig 6A) compared to longer ISIs (e.g., 900 ms; Fig 6B). We quantified the magnitude of response adaptation by measuring the reduction in the model response to T2 after subtracting out the response to T1 (i.e., the shaded blue region between curves in Fig 6A and 6B; [28,31]. With increases in either *τ*_E_ or *τ*_S_, response adaptation persisted across longer ISIs (Fig 6C and 6D), as longer time constants allowed the excitatory and/or suppressive drives to extend across longer ISIs, leading to normalization of T2 by T1. For combinations of shorter and longer time constant parameters (Fig 6E), suppression accumulated as both *τ*_E_ and *τ*_S_ increased. Thus, either excitatory temporal windows, suppressive temporal windows, or both could generate response adaptation, and had quantitatively similar effects on suppression indices.

These simulations also reveal that responses to repeated stimuli can merge together when the two stimulus presentations drive the same neural populations, particularly when ISIs are short (as in Fig 6A) and stimulus presentations are brief. In such situations, the sensory response can appear more like an extended response to one stimulus, reflecting temporal integration introduced by the excitatory and suppressive temporal windows. The degree of overlap in sequential responses may be related to perceptual integration across time, as previously theorized [34].

### Spatiotemporal normalization captures phenomena not explained by spatial or temporal normalization alone

Whereas the above results could be achieved with existing models that implement either just spatial or just temporal normalization, several neural phenomena involve interactions between space (or features) and time. Here we tested whether DSTN, with its unified spatiotemporal normalization computation, could capture such phenomena. For these purposes, features like orientation are treated equivalently to space, as in previous static normalization models [19]. The following analyses demonstrate how spatiotemporal normalization can generate suppression across stimuli with different feature values even when they are presented at different points in time. This property allows DSTN to exhibit neural and behavioral effects found empirically but not seen in existing normalization models. As these next analyses show, a notable consequence of spatiotemporal normalization is that it leads to normalization-linked suppression both forward and backward in time.

#### Response adaptation by non-identical stimuli.

**Fig 7:**
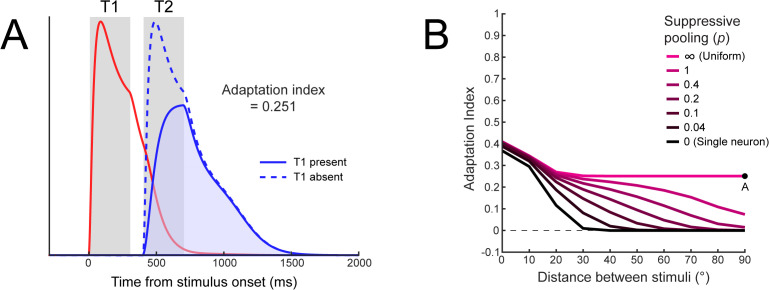
Response adaptation for non-identical stimuli in DSTN. **A)** Model sensory responses as a function of the presence of a preceding stimulus with an orientation difference of 90° and interstimulus interval of 100 ms, using *τ*_E _= 100 ms, *τ*_S _= 50 ms, and uniform suppressive pooling. Response adaptation is demonstrated by the reduction in T2 responses while T1 is present (shaded blue vs. dotted blue region). **B)** Effect of tuned suppressive pooling on response adaptation for non-identical stimuli. When the suppressive drive was pooled uniformly, adaptation persisted over large orientation differences between the adapter and test stimuli. As the pooling tuning width decreased, adaptation decreased for more dissimilar stimuli. Adaptation corresponding to panel A is indicated by the black dot.

Empirical work has shown that response adaptation can occur even with non-identical stimuli [35,36], with one study in particular finding that most MT neurons are adapted by a wider range of motion directions than they are responsive to [36]. This is a crucial observation, as neurons that are suppressed only by their own past activity, as in delayed normalization (DN) models [14,15], could not be suppressed by past stimuli that do not drive the neuron itself. In addition, neurons generally undergo less adaptation as the similarity between the adapting and test stimulus decreases [31,35,37]. Therefore we measured model responses to a preferred target stimulus while varying the orientation of the adapting stimulus and calculated an adaptation index (see Methods; Fig 7A).

We found, first, that adaptation was always strongest when the adapting stimulus and test stimulus matched in orientation. This property was due to the excitatory tuning of the model neurons: excitatory drives accumulated across both stimulus presentations when the neuron was sensitive to the adapting stimulus (<~20° from the preferred orientation), resulting in stronger normalization of the test stimulus. Second, adaptation by non-identical stimuli depended on the tuning of the suppressive pool. When suppression was uniformly pooled across all sensory neurons (magenta line in Fig 7B), adaptation remained relatively stable across larger differences between adapting and test stimuli. However, when suppression was adjusted to more strongly weight neurons tuned to similar orientations by changing the tuning width of the suppressive pool, adaptation progressively decreased and was even eliminated for more dissimilar orientations [38]. In the limit, when a neuron was only suppressed by its own activity—as in DN models [14,15]—adaptation occurred only for a narrow range of similar orientations. The ability to model different suppressive tuning profiles therefore allows DSTN another way to capture effects that depend on more complex spatiotemporal interactions, compared to previous models.

#### Backward masking.

**Fig 8:**
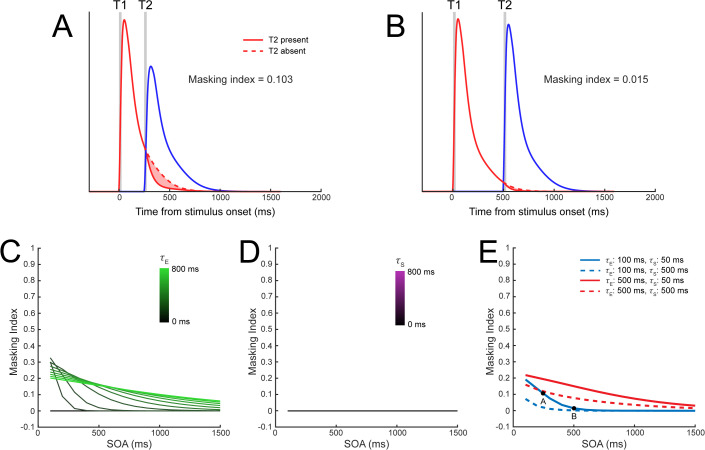
Backward masking requires excitatory temporal windows. **A)** Model sensory responses for one stimulus (T1, red lines) as a function of the presence of a subsequent stimulus (T2, blue line) with a stimulus onset asynchrony of 250 ms, using *τ*_E _= 100 ms and *τ*_S _= 50 ms. Backward masking is demonstrated by the reduction in T1 responses following the onset of T2, as indicated by the shaded red region. **B)** Backward masking was eliminated when the SOA was increased to 500 ms. **C)** Effect of *τ*_E_ and **D)** *τ*_S_ on backward masking for different SOAs. **E)** Combining different values of the *τ*_E_ and *τ*_S_ affected the pattern of backwards masking. Parameters corresponding to the simulations in panels A and B are indicated by labeled black dots on the solid blue line.

In backward masking, a neuron’s ongoing response to an initial stimulus is suppressed by a subsequent stimulus [7,39,40]. Similar to response adaptation, the magnitude of backward masking diminishes as the stimulus onset asynchrony (SOA) between stimuli increases. DSTN exhibited both backward masking and this characteristic SOA dependence, with greater masking at shorter SOAs (e.g., 250 ms; Fig 8A) than longer SOAs (e.g., 500 ms; Fig 8B). We simulated backwards masking using a sequence of two orthogonal stimuli and quantified the degree of masking in model simulations by measuring the response to T1 when T2 was present versus absent. Notably, the excitatory temporal window was necessary to produce backward masking, masking strength depended on *τ*_E_ (Fig 8C). Longer time constants resulted in backward masking that was generally stronger and persisted across longer intervals. In contrast, masking did not occur with the suppressive window alone, when *τ*_E_ was zero (Fig 8D). In this case, there was no temporal overlap in sensory responses to the two stimuli, which meant no normalization of the first stimulus by the second. However, when we explored the interaction between parameters, both *τ*_E_ and *τ*_S_ affected the magnitude of backward masking (Fig 8E). In general, higher *τ*_E_ values resulted in stronger backward masking, particularly for shorter SOAs (red versus blue lines in Fig 8E), as the extended sensory responses resulted in more overlap between the two stimuli and increased the overall normalization. In contrast, higher *τ*_S_ values resulted in reduced backward masking (dashed versus solid lines), as longer suppressive temporal windows tended to reduce the overlap in sensory responses between stimuli (cf. Fig 4D). Thus, the excitatory temporal window was necessary to produce backward masking, but so long as it was present, the strength of masking could be modulated by the suppressive temporal window.

#### Contrast-dependent suppression from both past and future stimuli.

A hallmark of spatial normalization models is that they generate suppressive effects that systematically depend on stimulus contrast [13,19]. Therefore we next investigated the contrast dependence of temporal context effects produced by DSTN. Specifically, we examined how model responses are affected by the contrast of a competing stimulus. In DSTN, contrast increases the magnitude of stimulus input, and thus increases the overall excitatory and suppressive drives in the sensory layers. As such, we expected contrast modulations to have effects similar to our simulations of response adaptation and backward masking: higher contrast (or presence versus absence) for one of two stimuli in a sequence would result in reduced model responses to the other.

**Fig 9:**
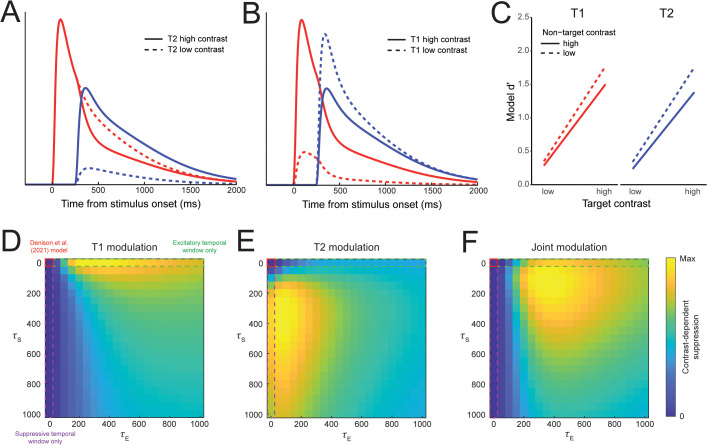
Contrast-dependent suppression forward and backward in time. **A)** When T1 (red lines) was fixed at high contrast, its response was modulated by the contrast of T2 (red solid line vs. dashed line), using *τ*_E _= 400 ms and *τ*_S _= 100 ms. **B)** Likewise, the response to a high contrast T2 was modulated by the contrast of T1 (blue solid vs. dashed lines). **C)** Modeled behavioral prediction in terms of *d*′ for discriminating clockwise vs. counterclockwise stimulus orientations. Performance was lower for each target stimulus when the non-target was presented at higher contrast, consistent with recent empirical observations. **D)** Effect of *τ*_E_ and *τ*_S_ on the contrast-dependent modulation of T1, **E)** T2, and **F)** the joint modulation of both stimuli, calculated by a pointwise multiplication of individual target heatmaps. A region of the parameter space with moderate *τ*_E_ and low *τ*_S_ provided the strongest combined modulation of both target stimuli, comparable to human behavior [41].

We first simulated model responses using an SOA of 250 ms, with excitatory and suppressive time constants that resulted in both response adaptation and backward masking (*τ*_E_ = 400 ms, *τ*_S_ = 100 ms; see Fig 2C). When the contrast of the second stimulus (T2; Fig 9A) was high (64%) versus low (16%) we observed a reduction in the model response to the first stimulus (T1). Likewise, when the contrast of T1 was high versus low, the model response to T2 was reduced (Fig 9B). Both effects were due to increases in the overall suppressive drive caused by higher contrast stimuli, resulting in stronger normalization of the model response to the other stimulus.

Psychophysical work has demonstrated that orientation discriminability for a target stimulus can be impaired by a non-target stimulus presented either before or after it by 250 ms, with greater impairment for higher versus lower contrast non-target stimuli [41]. To determine if the model could also produce this behavioral pattern, we used the decision layer of DSTN to calculate discriminability (*d*′) between different target orientations (Fig 9C). Model *d*′ was higher when the non-target was presented at a lower contrast, regardless of the target contrast. Notably, this effect occurred both forward (i.e., the contrast of T1 affected responses to T2) and backward in time (i.e., the contrast of T2 affected responses to T1). In these simulations, excitatory drives were affected by target contrast, with higher target contrast resulting in stronger sensory responses and higher model *d*′ for targets. On the other hand, the effects of non-target contrast were imparted through the suppressive drive, with high contrast non-targets resulting in lower sensory responses and *d*′ for targets.

We also explored how the excitatory and suppressive temporal windows modulated the effects of one stimulus’s contrast on the model responses to the other stimulus. We found that temporal windows were necessary for contrast-dependent suppression, as when both time constants were zero—reducing the model to the version in [18]—there was no modulation for either stimulus (Fig 9D–9F, red solid outline). For T1, we found that contrast-dependent suppression was strongest for a wide range of *τ*_E_ values (~250–850 ms) combined with a low *τ*_S_ (0–100 ms; Fig 9D). In particular, we found that suppression alone (i.e., *τ*_E _= 0) was insufficient to produce the observed effects (Fig 9D, purple dashed outline), because the lack of any extended excitatory drive meant the sensory responses did not overlap in time. This is comparable to the lack of backward masking present in our simulations manipulating only *τ*_S_ (cf. Fig 8D). In contrast, modulation of T2 was strongest when *τ*_S_ was an intermediate value (~250–650 ms) and *τ*_E_ was low but non-zero (~50–150 ms; Fig 9E). To assess what temporal window parameter combinations produced contrast-dependent suppression simultaneously across both T1 and T2 (Fig 9F), we computed a combined modulation index, which showed a range of intermediate *τ*_E_ values (300–600 ms) combined with low *τ*_S_ (50–200 ms) that resulted in moderate suppressive effects for both T1 and T2 (as in Fig 9C, with *τ*_E _= 400 ms and *τ*_S _= 100 ms). Notably, these same parameter ranges also reproduce the full set of phenomena we explored here. The ability of DSTN to produce bidirectional contrast-dependent suppression demonstrates how temporal normalization can account for interactions between stimuli that are separated in time, both affecting ongoing sensory processing as well as behavioral responses.

## Discussion

Normalization is a successful computational principle that predicts neural responses and perception, yet most previous models have considered normalization across space or time alone. Here, we demonstrate how spatiotemporal normalization can be implemented in a neural network model of dynamic visual processing. DSTN is grounded in the framework of normalization established by Reynolds and Heeger [19], which was subsequently extended into a dynamic spatial normalization model by Denison, Carrasco, and Heeger [18]. The core of DSTN is a unified spatiotemporal normalization computation, which provides local contextual modulation across space, time, and features. This normalization computation is supported by spatiotemporal receptive fields, with excitatory and suppressive drives that depend on recent stimulus history. This architecture generalizes across three classes of previous models: 1) static spatial normalization models [19], which lack a temporal dimension; 2) delayed normalization models [14,15], which include temporal normalization but lack spatial and featural dimensions, and; 3) the normalization model of dynamic attention [18], a dynamic model which includes normalization across space and features, but lacks temporal normalization. Spatiotemporal normalization in DSTN encompasses all these previous models, while also going beyond their simple combination, allowing DSTN to generate additional phenomena not previously captured.

We report several findings. First, the model sensory neurons exhibit temporal receptive fields with biphasic profiles, similar to neuronal receptive field properties observed empirically [20]. Notably, this biphasic profile was not incorporated directly into the model but emerged from the interaction between the excitatory and suppressive drives, the exponential temporal windows, and the normalization computation. Second, DSTN exhibited surround suppression, consistent with static spatial normalization models [12]; and it reproduced several non-linear dynamic response properties, including transient-sustained response dynamics [27,28], subadditivity with increasing stimulus duration [14,29], and response adaptation/repetition suppression [30–32], consistent with previous delayed normalization models [14,15]. Third, DSTN goes beyond both spatial and delayed normalization models to predict a range of phenomena that depend on spatiotemporal or feature-temporal interactions: adaptation by non-identical stimuli [36], backward masking [7,39,40], and bidirectional contrast-dependent suppression between successive stimuli [41]. DSTN can thus account for a wide range of neural and behavioral findings in the domain of dynamic vision through spatiotemporal normalization.

We implemented spatiotemporal normalization in DSTN by allowing the excitatory and suppressive drives to depend on recent stimulus input via their “temporal windows,” the temporal component of their spatiotemporal receptive field structure. These temporal windows cause the excitatory and suppressive drives to persist within the model sensory layers even after a stimulus has offset, allowing for normalization to occur between stimuli that are presented at distinct points in time. We found that the excitatory and suppressive temporal windows contributed to the observed phenomena in different ways. Increasing *τ*_E_ carried neural responses forward in time, after stimulus input had ended (Fig 4A), such that these responses could suppress (and be suppressed by) responses to subsequent stimuli. This was necessary for reproducing suppression backward in time (i.e., backward masking or contrast-dependent suppression from a subsequent stimulus), because responses needed to be carried forward across ISIs so that normalization could occur between neurons tuned to different features. In contrast, increasing *τ*_S_ allowed suppressive drives to accumulate across longer intervals, increasing the overall suppression of responses to prolonged stimulus presentations, resulting in transient-sustained responses typical of neural firing (Fig 4D), but also enabling suppression to be carried forward after responses to an initial stimulus have ended. This extended suppressive drive was sufficient to reproduce suppressive effects forward in time (i.e., response adaptation and contrast-dependent suppression from a preceding stimulus). For phenomena isolated within a single neuron (e.g., subadditivity, response adaptation by an identical stimulus) the excitatory temporal window reproduced equivalent patterns since history-based changes in the excitatory drive propagate to the suppressive drive, even when suppression is only instantaneous (*τ*_S _= 0 ms). Thus, the excitatory and suppressive temporal windows had distinct effects on neural timecourses, resulting in different patterns of effects across the phenomena we investigated. Importantly, combining both the excitatory and suppressive temporal windows in DSTN was necessary to produce this wide array of phenomena. We observed that contrast-dependent suppression was strongest across both stimuli for a range of intermediate excitatory values (*τ*_E _= 300–600 ms) and low suppressive values (*τ*_S _= 50–200 ms), and a model neuron with time constants within this range produce the other phenomena we examined as well. While all sensory neurons shared the same values for *τ*_E_ and *τ*_S_ within a given simulation, it is likely that that within regions of visual cortex neurons have some variety in their temporal windows, evidenced by the heterogeneity of neural response dynamics in electrophysiological recordings [21,28,42]. Therefore, allowing the time constants to vary within a population may better capture these previous empirical findings. In future work it will be interesting to investigate how diversity in response dynamics across individual neurons affects the response dynamics of the population and whether such diversity provides any computational benefits.

Other models have attempted to account for several of the dynamic neural response properties we investigated here. Delayed normalization (DN) models, for example, divide a neuron’s excitatory drive by a filtered and delayed copy of itself, generating neural response time courses that can be fit to observed fMRI [29] or electrocorticography data [14,15], producing transient-sustained responses, subadditivity, and response adaptation. Compressive spatiotemporal (CST) models fit separate sustained and transient channels that are passed through a compressive nonlinearity to produce responses [16,17]. Although CST models have not been tested for the same set of response dynamics, they are well fit to fMRI BOLD time courses in response to sequences of stimuli with different spatial locations and timings, and they reproduce increasing temporal window sizes along the visual hierarchy [5,6]. In both types of models, normalization is computed independently within each recorded unit (voxel, electrode site, single-unit, etc.). In contrast, DSTN implements suppressive pooling, both spatially—via summation across model neurons within layers—and temporally—as a consequence of the temporal windows—such that normalization can be induced by activity generated by other units and at other times. Pooling suppression across neurons is a property key to previous foundational normalization models [19] and allows for stimuli outside of the “classical receptive field” (i.e., stimuli that do not by themselves drive a neuron) to suppress a neuron’s response. Spatial pooling allows DSTN to capture temporal phenomena that are not necessarily localized to a single unit, such as backward masking, contrast-dependent suppression, and response adaptation to non-identical stimuli. Additionally, while DN and CST models both produce continuous neural time courses, these are computed based on *a priori* knowledge of the full stimulus sequence. DSTN, in contrast, produces layer responses in a recursive manner, with computations implemented “online” at each timestep.

Other models have implemented normalization in a dynamic framework. Louie and colleagues [43] modeled decision circuits in LIP using excitatory and inhibitory model neurons, noting that the recurrent interaction between neurons resulted in normalization that depended on an exponential weighting of previous excitatory activity. The suppressive temporal window in DSTN matches with this formulation, demonstrating that temporal normalization can explain sensory phenomena, as well as the dynamics of decision-making. More recently, Ernst and colleagues [42] used a model similar to that of [43] to predict a variety of transient-sustained responses recorded from MT neurons. Their dynamic equations used separate time constants for updating the excitatory and inhibitory responses over time, and notably in almost all neurons the fitted inhibitory time constant was longer than excitatory time constants. In DSTN, this property—slower suppression than excitation—is a necessary consequence the model structure, since suppressive drives depend on both *τ*_E_ and *τ*_S_, so even when *τ*_S_ is shorter than *τ*_E_, the effective time constant of suppression is longer than τ_E_. It remains to be determined whether this is indeed a universal property in cortex, as predicted by DSTN.

Spatial normalization across local neuronal populations has been proposed as a canonical computation in the brain that may provide benefits to coding efficiency [12,44], and evidence for it has been found in a variety of cortical regions [26,45–49]. In a similar way, temporal normalization may enhance sensitivity to changes in the environment, with transient-sustained dynamics, subadditive neural responses, and response adaptation demonstrating that consistent inputs act to reduce overall neural activity [15,29]. Temporal normalization may also emphasize differences between stimuli across time, as in the contrast-dependent suppression effects we observed, where model *d*′ was increased for high contrast targets among low contrast non-targets. Notably, previous formulations of temporal normalization capture forward suppression (e.g., adaptation) but not backward suppression, but coding efficiency should benefit from reducing redundancies bidirectionally in time. DSTN provides such a bidirectional normalization mechanism. These effects on coding efficiency also depend on how suppression is pooled across a neural population, as we observed when we manipulated suppressive tuning width during response adaptation: if suppression more strongly weighs inputs from similarly tuned neurons (i.e., smaller *p* in Fig 7B), it can increase sensitivity to larger changes in stimulus features over time. Conversely, broader tuning offers robustness against small perturbations caused by noise and can promote stability of representations over time [38,50].

DSTN is a flexible model that can be extended in different ways. First, the model can handle different spatial and feature dimensions through the tuning properties of the sensory neurons. In the current study, we manipulated the spatial tuning of sensory layer neurons to examine surround suppression and the tuning of the suppressive pool to investigate feature-tuned adaptation. DN models, in which suppression is calculated separately for each neural unit [14,15], are essentially a special case of tuned suppression, where the suppressive pool is tuned narrowly to one neuron. Second, we defined the excitatory and suppressive temporal windows using exponential functions, but different parameterizations are possible. Third, it may be interesting to examine how the modeled excitatory and suppressive time constants relate to the dynamics of excitatory and inhibitory neurons within different neural circuits. Computational work has demonstrated how normalization can be implemented through recurrent excitation and inhibition [51], providing a class of models with which to examine dynamic processing at the level of cortical circuits. Fourth, DSTN can be expanded to include multiple hierarchical sensory layers with different spatiotemporal receptive fields and pooling profiles. Neural dynamics vary between different regions, and temporal integration windows have been found to increase along the cortical hierarchy [2,4–6,52,53], suggesting that temporal windows may be successively applied and accumulate over several stages of processing. Varying the temporal parameters in DSTN across layers may therefore allow it to capture this increase in temporal integration along the visual hierarchy, and fitting DSTN to neural data could allow quantitative characterization of spatiotemporal receptive fields across the visual cortical hierarchy. Finally, normalization has been closely linked to attention, with normalization-based models of spatial [19,54,55], feature-based [56], object-based [57], and temporal attention [18] accounting for changes in behavior and neural responses. A natural extension to DSTN will be to examine how attention interacts with spatiotemporal normalization to influence ongoing processing.

DSTN makes several novel, testable predictions for future empirical work:

1. DSTN makes the strong prediction that single-neuron response dynamics can be parsimoniously characterized by two excitatory and suppressive temporal window parameters, *τ*_E_ and *τ*_S_, such that for a given neuron, a single set of such parameters should capture the neuron’s response dynamics in the wide variety of spatiotemporal phenomena we explored here. To test this prediction, temporal window parameters for a given neuron can be fit using one stimulation protocol and tested in other protocols to see if they correctly predict the neuron’s response dynamics. For example, the temporal window parameters fit for a neuron using reverse correlation should directly predict both the strength and time course of that neuron’s subadditivity, response adaptation, backward masking, etc. A finding that a single set of temporal parameters generalizes across these different phenomena for a given neuron would support DSTN, whereas failure to generalize would contradict model predictions. It is also possible that parameter generalization would hold for some visual areas but not others, which would provide insight into the computations supporting dynamic stimuli across the visual hierarchy.
2. DSTN predicts that biphasic temporal receptive fields can emerge from measuring responses to continuous visual stimuli, as in reverse correlation protocols, even when a neuron does not exhibit an inhibitory undershoot following the excitatory response to a single stimulus presentation. According to the model, such behavior could occur because receptive fields arise from divisive suppression of past time points rather than subtractive inhibition. Finding that a neuron has a biphasic receptive field when measured with reverse correlation but does not exhibit an inhibitory undershoot to single stimulus presentations would therefore support DSTN, whereas only finding that these phenomena co-occur would challenge it. Reverse correlation techniques have been used to map spatiotemporal receptive fields in mammalian LGN [20,21], V1 [58], and MT [22], as well as in visual regions in Drosophila [59], which may have allowed biphasic temporal receptive fields to be revealed in these cases. Although spatiotemporal receptive fields have mostly been investigated for their relevance to motion perception, our findings suggest that they may play a much broader role in visual processing.
3. Because suppressive spatiotemporal windows depend on excitatory drives that have already been filtered through excitatory spatiotemporal windows (Fig 1B), DSTN predicts that the resulting suppressive fields governing normalization must be broader than their excitatory field counterparts, both spatially and temporally (Fig 1A). Following from this principle, DSTN predicts that neurons will exhibit adaptation to stimuli that fall outside their classical receptive fields. There is some extant data supporting this prediction in the context of tuning for motion direction [31], but more systematic characterization of the spatiotemporal structure of suppressive fields together with the properties of adaptation for the same neurons will be needed to test whether this core prediction of DSTN holds more generally. A related open question amenable to empirical measurements is whether spatial and temporal suppressive field components are independent—with the spatial tuning of suppression constant across time, as we have assumed here—or interacting, such that the spatial tuning of suppression changes (likely, narrows) further into the past.
4. DSTN also makes behavioral predictions that can be tested with human participants. Here we found that DSTN reproduced an empirical finding of bidirectional contrast-dependent suppression between stimuli with a 250 ms SOA [41]. DSTN predicts that the magnitude of contrast-dependent suppression should decrease when stimuli are separated further in time, consistent with interval dependency of adaptation and backward masking. Based on simulations using parameters that captured the relative magnitudes of contrast-dependent suppression for the two targets in the empirical data (*τ*_E _= 400 ms, *τ*_S _= 100 ms), the model predicts that contrast-dependent suppression will be eliminated at SOAs of 700–1,000 ms. This quantitative prediction can be readily tested behaviorally. If supported, it would bolster the idea that contrast-dependent suppression is a consequence of spatiotemporal normalization, linking behavioral findings to neural processes. On the other hand, the model would be challenged if it were unable to fit behavioral contrast-dependent suppression data across a range of SOAs with a single set of temporal window parameters.

In summary, we introduced the dynamic spatiotemporal attention and normalization model, DSTN. The model integrates standard spatial and feature-based neural tuning functions with excitatory and suppressive temporal windows to generate a unified spatiotemporal normalization computation, resulting in a spatiotemporal receptive field structure like that seen in physiological recordings of sensory neurons. DSTN reproduces several non-linear response properties, including subadditive responses, response adaptation, and backwards masking, as well as contrast-dependent suppression between stimuli across time, a perceptual phenomenon only recently shown in human observers [41]. Our model provides advances over other dynamic normalization [14,15,18] or CST [16,17] models, such as the recursive neural network architecture that allows for continuous online prediction of layer responses, as well as the flexibility afforded by the temporal window and suppressive pooling structures, which allows both spatial and temporal normalization to be carried out via a single, parsimonious spatiotemporal computation. Overall, DSTN provides a step toward the goal of developing real-time process models of dynamic vision.

## Materials and methods

### Model specification

DSTN is a hierarchical, recurrent neural network, which models the dynamics of feature-tuned neural populations given time-varying sensory input. DSTN consists of interconnected sensory and decision layers, that each produce time-varying responses in model neurons, allowing the model to generate predictions about neural activity from continuous input in an online fashion (i.e., time step by time step) as well as to generate predictions about behavioral performance in simple perceptual tasks (Fig 1). DSTN is built on a modeling foundation established by Denison, Carrasco, and Heeger [18] and Reynolds and Heeger [19]. The introduction of excitatory and suppressive temporal windows together with a spatiotemporal normalization computation allows DSTN to generate a rich repertoire of dynamic behavior and to exhibit effects of temporal context in line with empirical observations, which could not be generated by these previous models. DSTN also contains voluntary and involuntary attention layers [18], though these were removed for all analyses in the current study.

#### Sensory layer.

The sensory layer represents the visual processing stage of the model. As in the normalization model of dynamic attention [18], this layer receives stimulus input at each time point, which feeds into *N* = 12 neurons that are tuned to different feature values. We use orientation as an example feature throughout the reported analyses. In simulations, the time course of stimulus input was represented in the matrix ***X*** (with size of M orientations × K time points), with each vector ***X***_***t***_ = (0, 0, …, *c*, …, 0) indicating the currently presented orientation at contrast level *c*. When no stimulus was presented, all elements of the vector were zero. The stimulus drive to each neuron at each time point depended on the match between the stimulus orientation and the neuron’s orientation tuning function, as determined using a raised cosine function:


$$\begin{array}{c} d_{it}=\;\left|{\text{cos}(\theta_{t}-\varphi_{i})}^{m}\right|\cdot c \end{array}$$ (1)

where $\theta_{t}$ is the orientation of the stimulus shown at time *t*, $\varphi_{i}$ is the preferred orientation of the *i*th neuron, and *c* is the stimulus contrast. Orientation tuning functions were evenly spaced across the feature space, $\varphi_{i}=\pi (i-\text{1})/N$. The exponent *m* controls the width of the tuning curve and was set to 23 (*m* = 2*N* − 1).

Unlike in previous models, the excitatory drive for each neuron is calculated at each time point using a weighted exponential of recent stimulus drive:


$$\begin{array}{c} e_{it}=\sum_{T=\text{0}}^{t}d_{iT}^{n}\cdot w_{T}^{E} \end{array}$$ (2)

$$\begin{array}{c} w_{T}^{E}=\frac{\text{1}}{\tau_{E}}e^{\frac{T-t}{\tau_{E}}} \end{array}$$ (3)

where *t* is the current time point, n is an exponent that affects the shape of neurons’ contrast response functions, and *τ*_E_ is the time constant determining the amount of weight given to previous time points, which was set to the same value for all model neurons. In effect, this excitatory temporal window imbues the model neurons with a temporal receptive field, where not only are units responsive to their preferred stimulus at any given time point, but also respond based on the stimulus history as well.

The suppressive drive was also calculated at each time point, and weighted by an exponential:


$$\begin{array}{c} s_{t}=\sum_{i}\sum_{T=\text{0}}^{t}e_{iT}\cdot w_{T}^{S} \end{array}$$ (4)

$$\begin{array}{c} w_{T}^{S}=\frac{\text{1}}{\tau_{S}}e^{\frac{T-t}{\tau_{S}}} \end{array}$$ (5)

where *τ*_S_ is the time constant determining the amount of weight given to excitatory drives at previous time points, which was set to the same value for all model neurons. Thus, a neuron’s current response is suppressed by the activity history of itself and its neighbors, which together comprise the suppressive pool. Unless otherwise specified, only a single spatial location was modeled, and neurons tuned to all orientations were included with equal weight in the suppressive pool. But in principle, the suppressive pool could also be tuned to locations and features.

Finally, the response of each neuron was updated at every time step using the following differential equation, which functions as a dynamic update of the standard normalization equation (19), as in the normalization model of dynamic attention [18]:


$$\begin{array}{c} \tau_{R}\frac{d}{dt}r_{i}=-r_{i}+\frac{e_{i}}{s+\sigma^{n}} \end{array}$$ (6)

where *r*_*i*_ is the response of the *i*th neuron within the sensory population, *τ*_*R*_ is a time constant that affects the rate at which the neuron’s response increases during stimulus presentation and decreases after stimulus offset, *e*_*i*_ is the excitatory drive of that neuron, s is the (pooled) suppressive drive, *σ* is a semi-saturation constant that affects the contrast gain of neurons and keeps the denominator non-zero, and n is an exponent following equation (2). Some parameter values (*τ*_*R*_ = 52, *n* = 1.5) were fixed as fitted to empirical data in previous reports [18], however we decreased the semi-saturation constant (*σ* = 0.1) to account for the fact that we only used a single sensory layer in this version of DSTN.

#### Decision layer.

The decision layer receives input from the sensory layer, which it uses to compute behavioral output regarding the orientation of the stimuli, as in the normalization model of dynamic attention [18]. Decisions are generated by two neurons, each encoding for one of the two stimuli (T1 and T2). The excitatory drive $e_{jt}^{D}$ for each decision neuron is:


$$\begin{array}{c} e_{jt}^{D}=r_{t}^{S}\cdot w_{j}^{D} \end{array}$$ (7)

where each weight vector $w_{j}^{D}$ is designed to compute an optimal linear readout from the sensory responses $r_{t}^{S}$ to the *j*th stimulus, to determine whether the stimulus was rotated clockwise versus counterclockwise from horizontal or vertical. The vectors project the sensory layer response onto the difference between two templates encoding the population responses to the CW and CCW stimuli along a given orientation axis; the axis of each stimulus (vertical or horizontal) is assumed known. Evidence accumulation is positive for CW decisions, and negative for CCW decisions, such that the sign of the evidence indicates the decoded orientation within the decision layer, while the magnitude of the evidence indicates the strength of the model decision. The suppressive drive is pooled over the decision neurons, and responses are updated at each time point according to the same differential equation as in sensory layers (*τ*_*D*_ = 10^5^, *σ*_*D*_ = 0.7) [18]. The long time constant of this layer allows for sustained evidence accumulation.

In contrast to previous model iterations [18], each neuron in the decision layer accumulates evidence throughout the duration of the simulated trials, rather than during discrete windows following each stimulus presentation. Because the effects of temporal normalization occur when the excitatory and suppressive drives elicited by the two stimuli overlap, the discrete decision windows were unable to capture any behavioral effects of normalization for T1 at short SOAs. At the end of each simulation, the accumulated evidence for each stimulus is converted into *d*′, a measure of perceptual sensitivity, through multiplicative scaling (*s*_T1_ = *s*_T2_ = 1 × 10^5^).

### Simulation procedures

All simulations were performed in MATLAB (2022b, MathWorks, Natick, MA). We used a time step Δ*t* of 2 ms throughout simulations. The duration of the simulated trial was varied as necessary to capture model dynamics. When a stimulus was presented, we used a standard contrast level of 64% and presentation duration of 30 ms, unless otherwise specified. We varied the two temporal window time constants *τ*_*E*_ and *τ*_*S*_ across simulations to assess how the temporal windows affected sensory layer dynamics and decision layer outputs.

#### Estimating temporal receptive fields of model neurons.

To assess how stimulus input at different past time points affects model responses at the current time, we performed a simulation and analysis based on reverse correlation, similar to the way a temporal receptive field might be measured in a neurophysiology experiment [20]. We modified the stimulus input to the model to be a random binary vector, such that the stimulus drive at each time point was one or zero. We then performed model simulations for 10,000 different random stimulus vectors with a duration of 1,200 ms, resulting in variable excitatory and suppressive drives and sensory layer responses that depended on the stimulus history. To estimate the impact of the stimulus input on each model timeseries, we used reverse correlation to calculate the average change in each measure caused by the presence of a stimulus at each time point by correlating the stimulus input at each time point with the response (excitatory/suppressive drive or layer response) at the final time point [60]. Shuffled weights were calculated in the same manner after rearranging the stimulus input vectors so that they were not aligned with the responses from the same simulation.

To characterize the temporal receptive fields and enable comparisons across different parameter settings, we fit the estimated stimulus weights using different functional forms. For the excitatory drive, we compared the estimated stimulus weights with the exponential function that defines the excitatory temporal window. This function (*τ*_*E*_ = 400 ms) had no free parameters, but was adjusted using a scaling parameter to align it to the magnitude of the stimulus weights (the units of which are arbitrary), estimated using simple linear regression. For the suppressive drive, we similarly overlaid a scaled function to the stimulus weights. This function, *h*_*S*_, was determined by convolving the excitatory and suppressive temporal windows, using their respective time constants. We zero-padded the temporal windows at positive (i.e., future) time points to achieve the resulting functional form. Notably, this function in general can also be calculated by taking the difference between the exponential temporal windows (for *t* < 0 and *τ*_*E *_≠ *τ*_*S*_):


$$\begin{array}{c} h_{S}=\left|e^{\frac{t}{\tau_{E}}}-e^{\frac{t}{\tau_{S}}}\right| \end{array}$$ (8)

The sensory layer response weights were fitted with a difference of Gamma functions, using the simplified Gamma function form adopted in previous work [15]:


$$\begin{array}{c} h_{R}=te^{\frac{t}{\tau_{\text{1}}}}-kte^{\frac{t}{\tau_{\text{2}}}}\; \end{array}$$ (9)

with time constants *τ*_*1*_ and *τ*_*2*_, and weight *k*. We fitted the simulated stimulus weights to this function in MATLAB by minimizing the least-squares error using *fminsearch* up to a scaling factor. We performed this optimization 100 times, with random initial values for *τ*_*1*_ and *τ*_*2*_ drawn uniformly between 0 and 900, and initial *k* = 0, and selected the best fitting function across all solutions. For the function shown in Fig 2C, the best fitting parameters were: $\hat{\tau_{\text{1}}}=\text{3}\text{0}\text{5}.\text{0}\text{1},\;\hat{\tau_{\text{2}}}=\text{6}\text{1}.\text{9}\text{8},\;\hat{k}=\text{5}.\text{4}\text{3}$.

To assess how the effective temporal receptive fields were affected by the excitatory and suppressive temporal parameters, we performed the model simulations again for each combination of *τ*_*E*_ and *τ*_*S*_ from 100 to 900 ms in 100 ms steps. In the results shown in Fig 2D and 2E, we took the fitted functions for one parameter fixed at 400 ms, while the other parameter varied.

#### Surround suppression.

To confirm that DSTN maintains the spatial normalization properties observed in previous static models, we simulated how the model response to a stimulus was affected by a nearby stimulus presented outside its receptive field. To model the spatial dimension, we defined two populations of neurons each with feature tuning to multiple orientations, but tuned to distinct spatial locations. Whereas each subpopulation was excited only by stimuli in its preferred spatial location, suppressive drives were pooled across the two subpopulations (i.e., across space), allowing for mutually suppressive interactions between the two spatial locations. We assigned one subpopulation as “center” neurons, and the other as “surround” neurons. Across simulations, we varied the contrast of the center stimulus across a wide range of values (from 5% to 100% contrast at 20 levels, log-spaced) to map the contrast response function (CRF) of model neurons, and varied the contrast of the surround stimulus at fixed levels (0%, 12%, 25%, 50%, or 100%) to assess surround suppression. For each simulation, we measured the response in the neuron maximally responsive to the center orientation, summed across the timecourse of the simulation. We used a fixed set of time constants (*τ*_*E *_= 400 ms, *τ*_*S*_ = 100 ms), presenting the center and surround stimuli simultaneously for 100 ms. We also used a lower semi-saturation constant for this simulation (*σ* = 0.02) to produce CRFs that spanned the neuron’s response range. The implementation of just two adjacent spatial populations is a simplification compared to previous implementations of spatial normalization that model a continuous spatial map [19,25]. Nevertheless, this simplified implantation is sufficient to test whether the model can reproduce spatial normalization effects such as surround suppression, and it demonstrates how the proposed architecture can be flexibly expanded as needed to generate spatial and feature maps in the sensory layer.

#### Effects of temporal receptive fields on neural responses.

To assess the effects of the excitatory and suppressive time constants on the model neuron responses, we simulated trials in which a single target stimulus was presented. The stimulus presentation duration was set to 2,000 ms to allow time to observe the peak and steady state responses, and the total trial duration was set to 8,100 ms, including a 500 ms prestimulus period. We varied *τ*_*E*_ and *τ*_*S*_ separately across 9 levels (50, 100, 200, 300, 400, 500, 600, 700, and 800 ms). To examine the effects of the individual time constants in isolation, for simulations varying *τ*_*E*_, we fixed *τ*_*S*_ at zero, and vice-versa, effectively removing the excitatory or suppressive temporal receptive fields from the model. Setting *τ*_*E *_= *τ*_*S *_= 0 reduces the model to the previous version [18]. For these simulations, we extracted response time courses from the sensory layer neuron tuned nearest to the target orientation. We also examined the interaction of the time constants by conducting simulations with different combinations of non-zero values for *τ*_*E*_ and *τ*_*S*_.

For simulations varying the excitatory time constant, all model neurons reached the same stable activity level, and all neural responses were scaled by dividing the response at all time points by the maximum response within the stimulus presentation window. For each value of *τ*_*E*_, we found the time point at which the model responses reached 99% of the maximum response (“Time to peak”, Fig 3B), and the time point at which responses fell to 50% of the peak value following stimulus offset (“Time to half maximum”, Fig 3C). We used 99% of the maximum for the time to peak analysis, because responses approached but did not necessarily reach the numerical maximum until late in the window.

For simulations varying the suppressive time constant, we scaled all responses relative to the first peak (Fig 3D). Shorter suppressive time constants typically resulted in a faster reduction in model responses, reaching a peak sooner but also reducing the overall magnitude. To examine response dynamics relative to the peak response, we therefore normalized the response time course by the peak response during the stimulus presentation window. We calculated the time to peak by measuring the time at which the maximum response was reached (“Time to peak”, Fig 3E), as well as the model response 2000 ms after stimulus onset relative to the peak (“Stable activity level”, Fig 3F).

#### Subadditive responses.

To assess how neural responses depended on stimulus presentation duration, we again simulated trials with only a single target. For the simulations shown in Fig 5A, we used short time constants (*τ*_*E*_ = 100 ms, *τ*_*S*_ = 50 ms) and varied the stimulus duration by doubling from 30–480 ms, for 5 total duration conditions (30, 60, 120, 240, and 480 ms). We extracted responses from the sensory layer in the model neuron tuned closest to the target orientation, and calculated the model response by summing the sensory response across the entire trial duration, approximating the area under the curves in Fig 5A. To quantify subadditivity in neural responses, we calculated the effect of doubling the stimulus duration on the model response by dividing the response at duration 2x by the response at duration x (e.g., we divided the model response at 60 ms by that at 30 ms; Fig 5C). To assess how subadditivity depends on the excitatory and suppressive time constants, we selected one shorter and one longer value (*τ*_*E*_ = 100 or 500 ms, *τ*_*S*_ = 50 or 500 ms), and computed the proportional response change for each pair of time constants (Fig 5D).

#### Response adaptation.

Response adaptation refers to the finding that neural responses to stimuli presented shortly after an initial stimulus are typically reduced in magnitude [27,30–33]. In our analysis, we therefore aimed to examine how model responses differed to a sequence of two “target” stimuli (referred to as T1 and T2) relative to a single stimulus. We performed separate analyses where T1 and T2 were identical stimuli (i.e., the same feature) or distinct (i.e., orthogonal features). In the first simulation examining the interaction between responses to two identical stimuli, our goal was to assess the magnitude of the response to T2 while subtracting out the activity related to T1. Therefore, we first measured the response to T1 alone (Fig 6A, blue dashed line), and then quantified the activity related to T2 by calculating the difference in responses when T2 was present versus absent (Fig 6A, blue shaded region). We used a longer stimulus presentation duration of 300 ms, which is typical in the response adaptation literature [30]. For the simulation shown in Fig 6A and 6B, we again selected short time constants (*τ*_*E*_ = 100 ms, *τ*_*S*_ = 50 ms) and used an ISI of 100 ms. We extracted the sensory response in the maximally selective neuron to the stimulus in two simulations: 1) T2 present, 2) T2 absent. The simulation in Fig 6B was identical, except that the ISI was increased to 600 ms.

To quantify the effect of excitatory and suppressive time constants on the magnitude of response adaptation, we varied each of *τ*_*E*_ and *τ*_*S*_ separately across 10 levels (0, 50, 100, 200, 300, 400, 500, 600, 700, and 800 ms) while keeping the other time constant fixed at zero, and assessed the sensory response across a range of ISIs (100–1,500 ms, in 100 ms steps). To investigate whether temporal windows were required for response adaptation in this model, we also simulated a condition with *τ*_*E*_ and *τ*_*S*_ both equal to zero. For each value of the time constants, we performed simulations where T1 was present or absent. To isolate the response to T2 when T1 was present, we followed previous work [28,31] by first subtracting out the activity elicited by a single stimulus (here, when T2 was absent), as follows:


$$\begin{array}{c} AI=\text{1}-\frac{\sum_{t}\left(r_{T\text{2}present}-r_{T\text{2}absent}\right)}{\sum_{t}r_{T\text{2}absent}} \end{array}\;$$ (10)

where each *r* reflects the sensory layer response under a particular stimulus condition. A higher adaptation index corresponds to a smaller isolated T2 response and thus stronger response adaptation. To examine how the time constants interact to affect response adaptation, we conducted additional simulations using combinations of shorter and longer time constants (*τ*_*E*_ = 100 or 500 ms, *τ*_*S*_ = 50 or 500 ms), and computed suppression indices across the same range of ISIs (Fig 6E).

To assess the presence of response adaptation for non-identical stimuli (Fig 7), we performed further simulations in which we manipulated the difference in orientation between T1 (the adapting stimulus) and T2 (the test stimulus). In these simulations, we fixed the temporal window parameters (*τ*_*E*_ = 400 ms, *τ*_*S*_ = 100 ms) and the ISI (100 ms) to focus on the effects of feature similarity and suppressive pooling on the magnitude of adaptation. To manipulate the tuning of the suppressive pool, we computed a pooling matrix that weighs the influence of one neuron on another when calculating suppressive drives, as follows:


$$\begin{array}{c} S_{ij}=\;\left|{\text{cos}(\varphi_{i}-\varphi_{j})}^{\text{1}/p}\right| \end{array}$$ (11)

where *φ*_*i*_ and *φ*_*j*_ are the preferred orientations of the *i*th and *j*th neuron, respectively, and *p* is a scaling parameter that determines the sharpness of the suppressive pooling. In the limit when *p* = ∞, all weights are 1, producing uniform suppressive pooling. In contrast, when *p* is small, suppression is mostly driven by similarly-tuned neurons, and when *p* = 0 neurons are only suppressed by themselves.

Across simulations, we fixed the test stimulus orientation at 0° and varied the adapting stimulus orientation in 10° steps from 0° (identical) to 90° (orthogonal). We separately adjusted the suppressive tuning across simulations at 7 levels (*p* = ∞, 1, 0.4, 0.2, 0.1, 0.04, 0). For each set of parameters, we measured the response in the neuron tuned to the test orientation in three separate simulations presenting: 1) the test stimulus alone (*r*_test_); 2) the adapting stimulus alone (*r*_adapt_); 3) both the adapting and test stimulus (*r*_both_). We then calculated the (non-orthogonal) adaptation index, following Priebe and Lisberger (2002):


$$\begin{array}{c} AI=\text{1}-\frac{\sum_{t}r_{both}-r_{adapt}}{\sum_{t}r_{test}} \end{array}$$ (12)

#### Backward masking.

Backward masking refers to the phenomena that perception of a stimulus can be impaired (“masked”) by a second stimulus presented shortly after the first [7,39,40]. We assessed backward masking in the model using a similar method as for response adaptation, except with simulations comparing the sensory response to T1 as a function of whether T2 was present versus absent:


$$\begin{array}{c} \begin{array}{c} MI=\text{1}-\frac{\sum_{t}r_{T\text{2}present}}{\sum_{t}r_{T\text{2}absent}} \end{array} \end{array}$$ (13)

Again, we first conducted simulations using orthogonal stimulus orientations, with short time constants (*τ*_*E*_ = 100 ms, *τ*_*S*_ = 50 ms) and compared the effects of backward masking for SOAs of 250 ms (Fig 8A) and 500 ms (Fig 8B). We then quantified backward masking by calculating the response summed over time to T1 when T2 was present relative to when it was absent. We calculated this masking index across the full range of excitatory and suppressive time constants and SOAs (Figs 7C and 7D). Finally, we conducted additional simulations assessing the interaction of the two time constants on backward masking using combinations of one shorter and one longer time constant (*τ*_*E*_ = 100 or 500 ms, *τ*_*S*_ = 50 or 500 ms), and computed masking indices across the same range of SOAs (Fig 8E).

#### Contrast-dependent stimulus interactions.

We assessed how the contrast of one stimulus affects the response to the other stimulus through temporal normalization. For initial simulations, we used fixed time constants (*τ*_*E*_ = 400 ms, *τ*_*S*_ = 100 ms), with the SOA (250 ms) and stimulus contrasts (64% versus 16%) chosen to match the previous behavioral findings. We performed simulations independently manipulating the contrast of both T1 and T2 (64% or 16% contrast level), and extracted model performance for each target. We first examined how the model responses to each target at high contrast was affected by the contrast of the non-target (Figs 8A and 8B). We then computed the model’s behavioral discrimination performance (measured as *d*′) from the output of the decision layer, based on recent empirical findings showing that a high- versus low-contrast non-target stimulus presented before or after a target stimulus can result in reduced perceptual discriminability of the target [41]. Model responses were scaled to produce *d*′ values closer to behavioral estimates (*s*_T1_ = *s*_T2_ = 1 × 10^4^). Model *d*′ was calculated for each target stimulus based on the target and non-target contrast (Fig 9C).

To assess how contrast-dependent suppression for T1 and T2 was affected by the excitatory and suppressive temporal windows, we performed further simulations in which we varied *τ*_*E*_ and *τ*_*S*_ from 0 to 1,000 ms in 50 ms steps. For each simulation, we measured the model *d*′ for each target stimulus (T1 and T2) as a function of the contrast of the non-target (NT), as above. We then calculated a contrast-dependent suppression index for each stimulus as:


$$\begin{array}{c} SI=\frac{d_{NTlow}-d_{NThigh}}{d_{NTlow}+d_{NThigh}} \end{array}$$ (14)

To identify values of *τ*_*E*_ and *τ*_*S*_ that produced suppression in both T1 and T2, we calculated a joint suppression index across the two targets by multiplying the suppression indices of T1 and T2 for each parameter combination. This joint index is maximized when a specific combination of *τ*_*E*_ and *τ*_*S*_ results in contrast-dependent suppression in both stimuli, but not when one or both stimuli show little-to-no suppression.

## Abbreviations:

CRF: contrast response functions
CST: compressive spatiotemporal
DN: delayed normalization
DSTN: dynamic spatiotemporal normalization model
ISI: interstimulus interval
NT: non-target
SOA: stimulus onset asynchrony

## References

[1] Mauk MD, Buonomano DV. The neural basis of temporal processing. Annu Rev Neurosci. 2004;27:307–40. doi: 10.1146/annurev.neuro.27.070203.144247 15217335

[2] Fritsche M, Lawrence SJD, de Lange FP. Temporal tuning of repetition suppression across the visual cortex. J Neurophysiol. 2020;123(1):224–33. doi: 10.1152/jn.00582.2019 31774368 PMC6985851

[3] Hasson U, Yang E, Vallines I, Heeger DJ, Rubin N. A hierarchy of temporal receptive windows in human cortex. J Neurosci. 2008;28(10):2539–50. doi: 10.1523/JNEUROSCI.5487-07.2008 18322098 PMC2556707

[4] Gao R, van den Brink RL, Pfeffer T, Voytek B. Neuronal timescales are functionally dynamic and shaped by cortical microarchitecture. Elife. 2020;9:e61277. doi: 10.7554/eLife.61277 33226336 PMC7755395

[5] Murray JD, Bernacchia A, Freedman DJ, Romo R, Wallis JD, Cai X, et al. A hierarchy of intrinsic timescales across primate cortex. Nat Neurosci. 2014;17(12):1661–3. doi: 10.1038/nn.3862 25383900 PMC4241138

[6] Wolff A, Berberian N, Golesorkhi M, Gomez-Pilar J, Zilio F, Northoff G. Intrinsic neural timescales: temporal integration and segregation. Trends Cogn Sci. 2022;26(2):159–73. doi: 10.1016/j.tics.2021.11.007 34991988

[7] Breitmeyer BG, öǧmen H. Visual masking: time slices through conscious and unconscious vision. 2nd ed. Oxford, UK: Oxford University Press; 2006.

[8] Yeshurun Y, Rashal E, Tkacz-Domb S. Temporal crowding and its interplay with spatial crowding. J Vis. 2015;15(3):11. doi: 10.1167/15.3.11 25788705

[9] Cavanagh SE, Hunt LT, Kennerley SW. A diversity of intrinsic timescales underlie neural computations. Front Neural Circuits. 2020;14:615626. doi: 10.3389/fncir.2020.615626 33408616 PMC7779632

[10] Golesorkhi M, Gomez-Pilar J, Zilio F, Berberian N, Wolff A, Yagoub MCE, et al. The brain and its time: intrinsic neural timescales are key for input processing. Commun Biol. 2021;4(1):970. doi: 10.1038/s42003-021-02483-6 34400800 PMC8368044

[11] Soltani A, Murray JD, Seo H, Lee D. Timescales of cognition in the brain. Curr Opin Behav Sci. 2021;41:30–7. doi: 10.1016/j.cobeha.2021.03.003 34026949 PMC8136243

[12] Carandini M, Heeger DJ. Normalization as a canonical neural computation. Nat Rev Neurosci. 2011;13(1):51–62. doi: 10.1038/nrn3136 22108672 PMC3273486

[13] Heeger DJ. Normalization of cell responses in cat striate cortex. Vis Neurosci. 1992;9(2):181–97. doi: 10.1017/s0952523800009640 1504027

[14] Groen IIA, Piantoni G, Montenegro S, Flinker A, Devore S, Devinsky O, et al. Temporal dynamics of neural responses in human visual cortex. J Neurosci. 2022;42(40):7562–80. doi: 10.1523/JNEUROSCI.1812-21.2022 35999054 PMC9546476

[15] Zhou J, Benson NC, Kay K, Winawer J. Predicting neuronal dynamics with a delayed gain control model. PLoS Comput Biol. 2019;15(11):e1007484. doi: 10.1371/journal.pcbi.1007484 31747389 PMC6892546

[16] Kim I, Kupers ER, Lerma-Usabiaga G, Grill-Spector K. Characterizing spatiotemporal population receptive fields in human visual cortex with fMRI. Journal of Neuroscience. 2024;44(2). 10.1523/JNEUROSCI.0803-23.2023 PMC10866195 37963768

[17] Kupers ER, Kim I, Grill-Spector K. Rethinking simultaneous suppression in visual cortex via compressive spatiotemporal population receptive fields. Nat Commun. 2024;15(1):6885. doi: 10.1038/s41467-024-51243-7 39128923 PMC11317513

[18] Denison RN, Carrasco M, Heeger DJ. A dynamic normalization model of temporal attention. Nat Hum Behav. 2021;5(12):1674–85. doi: 10.1038/s41562-021-01129-1 34140658 PMC8678377

[19] Reynolds JH, Heeger DJ. The normalization model of attention. Neuron. 2009;61:168–85. 19186161 10.1016/j.neuron.2009.01.002 PMC2752446

[20] Mante V, Bonin V, Carandini M. Functional mechanisms shaping lateral geniculate responses to artificial and natural stimuli. Neuron. 2008;58(4):625–38. doi: 10.1016/j.neuron.2008.03.011 18498742

[21] Cai D, DeAngelis GC, Freeman RD. Spatiotemporal receptive field organization in the lateral geniculate nucleus of cats and kittens. J Neurophysiol. 1997;78(2):1045–61. doi: 10.1152/jn.1997.78.2.1045 9307134

[22] Perge JA, Borghuis BG, Bours RJE, Lankheet MJM, van Wezel RJA. Dynamics of directional selectivity in MT receptive field centre and surround. Eur J Neurosci. 2005;22(8):2049–58. doi: 10.1111/j.1460-9568.2005.04363.x 16262642

[23] Cavanaugh JR, Bair W, Movshon JA. Nature and interaction of signals from the receptive field center and surround in macaque V1 neurons. J Neurophysiol. 2002;88(5):2530–46. doi: 10.1152/jn.00692.2001 12424292

[24] Cavanaugh JR, Bair W, Movshon JA. Selectivity and spatial distribution of signals from the receptive field surround in macaque V1 neurons. J Neurophysiol. 2002;88(5):2547–56. doi: 10.1152/jn.00693.2001 12424293

[25] Carandini M. Receptive fields and suppressive fields in the early visual system. In: Gazzaniga MS, editor. Cognitive neurosciences. 3rd ed. Cambridge, MA: MIT Press; 2004. p. 313–26.

[26] Carandini M, Heeger DJ, Movshon JA. Linearity and normalization in simple cells of the macaque primary visual cortex. J Neurosci. 1997;17(21):8621–44. doi: 10.1523/JNEUROSCI.17-21-08621.1997 9334433 PMC6573724

[27] Motter BC. Modulation of transient and sustained response components of V4 neurons by temporal crowding in flashed stimulus sequences. J Neurosci. 2006;26(38):9683–94. doi: 10.1523/JNEUROSCI.5495-05.2006 16988039 PMC6674438

[28] Lisberger SG, Movshon JA. Visual motion analysis for pursuit eye movements in area MT of macaque monkeys. J Neurosci. 1999;19(6):2224–46. doi: 10.1523/JNEUROSCI.19-06-02224.1999 10066275 PMC6782544

[29] Zhou J, Benson NC, Kay KN, Winawer J. Compressive temporal summation in human visual cortex. J Neurosci. 2018;38(3):691–709. doi: 10.1523/JNEUROSCI.1724-17.2017 29192127 PMC5777115

[30] Vogels R. Sources of adaptation of inferior temporal cortical responses. Cortex. 2016;80:185–95. doi: 10.1016/j.cortex.2015.08.024 26518166

[31] Priebe NJ, Churchland MM, Lisberger SG. Constraints on the source of short-term motion adaptation in macaque area MT. I. The role of input and intrinsic mechanisms. J Neurophysiol. 2002;88(1):354–69. doi: 10.1152/jn.00852.2001 12091560 PMC2581621

[32] Kohn A. Visual adaptation: physiology, mechanisms, and functional benefits. J Neurophysiol. 2007;97(5):3155–64. doi: 10.1152/jn.00086.2007 17344377

[33] Solomon SG, Kohn A. Moving sensory adaptation beyond suppressive effects in single neurons. Curr Biol. 2014;24(20):R1012-22. doi: 10.1016/j.cub.2014.09.001 25442850 PMC4279707

[34] Akyürek EG. Temporal integration as an adaptive process in visual perception, attention, and working memory. Neurosci Biobehav Rev. 2025;170:106041. doi: 10.1016/j.neubiorev.2025.106041 39922439

[35] Liu Y, Murray SO, Jagadeesh B. Time course and stimulus dependence of repetition-induced response suppression in inferotemporal cortex. J Neurophysiol. 2009;101(1):418–36. doi: 10.1152/jn.90960.2008 18987118 PMC2637012

[36] Priebe NJ, Lisberger SG. Constraints on the source of short-term motion adaptation in macaque area MT. II. Tuning of neural circuit mechanisms. J Neurophysiol. 2002;88(1):370–82. doi: 10.1152/jn.2002.88.1.370 12091561 PMC2581620

[37] Patterson CA, Wissig SC, Kohn A. Distinct effects of brief and prolonged adaptation on orientation tuning in primary visual cortex. J Neurosci. 2013;33(2):532–43. doi: 10.1523/JNEUROSCI.3345-12.2013 23303933 PMC3710132

[38] Snow M, Coen-Cagli R, Schwartz O. Specificity and timescales of cortical adaptation as inferences about natural movie statistics. J Vis. 2016;16(13):1. doi: 10.1167/16.13.1 27699416 PMC5054764

[39] Enns J, Di Lollo V. What’s new in visual masking?. Trends Cogn Sci. 2000;4(9):345–52. doi: 10.1016/s1364-6613(00)01520-5 10962616

[40] Kovacs G, Vogels R, Orban GA. Cortical correlate of pattern backward-masking. Proc Natl Acad Sci U S A. 1995;92(12):5587–91. 7777553 10.1073/pnas.92.12.5587 PMC41741

[41] Epstein ML, Kovacevich S, Denison RN. Perceptual support for temporal normalization across hundreds of milliseconds. bioRxiv. 2025.

[42] Ernst UA, Chen X, Bohnenkamp L, Galashan FO, Wegener D. Dynamic divisive normalization circuits explain and predict change detection in monkey area MT. PLoS Comput Biol. 2021;17(11):e1009595. doi: 10.1371/journal.pcbi.1009595 34767547 PMC8612546

[43] Louie K, LoFaro T, Webb R, Glimcher PW. Dynamic divisive normalization predicts time-varying value coding in decision-related circuits. J Neurosci. 2014;34(48):16046–57. doi: 10.1523/JNEUROSCI.2851-14.2014 25429145 PMC4244470

[44] Louie K, Glimcher PW. Efficient coding and the neural representation of value. Ann N Y Acad Sci. 2012;1251:13–32. doi: 10.1111/j.1749-6632.2012.06496.x 22694213

[45] Busse L, Wade AR, Carandini M. Representation of concurrent stimuli by population activity in visual cortex. Neuron. 2009;64(6):931–42. doi: 10.1016/j.neuron.2009.11.004 20064398 PMC2807406

[46] Rabinowitz NC, Willmore BDB, Schnupp JWH, King AJ. Contrast gain control in auditory cortex. Neuron. 2011;70(6):1178–91. doi: 10.1016/j.neuron.2011.04.030 21689603 PMC3133688

[47] Simoncelli EP, Heeger DJ. A model of neuronal responses in visual area MT. Vision Res. 1998;38(5):743–61. doi: 10.1016/s0042-6989(97)00183-1 9604103

[48] Zoccolan D, Cox DD, DiCarlo JJ. Multiple object response normalization in monkey inferotemporal cortex. J Neurosci. 2005;25(36):8150–64. 16148223 10.1523/JNEUROSCI.2058-05.2005 PMC6725538

[49] Louie K, Grattan LE, Glimcher PW. Reward value-based gain control: divisive normalization in parietal cortex. J Neurosci. 2011;31(29):10627–39. doi: 10.1523/JNEUROSCI.1237-11.2011 21775606 PMC3285508

[50] Benucci A, Saleem AB, Carandini M. Adaptation maintains population homeostasis in primary visual cortex. Nat Neurosci. 2013;16(6):724–9. doi: 10.1038/nn.3382 23603708 PMC3665725

[51] Heeger DJ, Zemlianova KO. A recurrent circuit implements normalization, simulating the dynamics of V1 activity. Proc Natl Acad Sci U S A. 2020;117(36):22494–505. doi: 10.1073/pnas.2005417117 32843341 PMC7486719

[52] Chaudhuri R, Knoblauch K, Gariel M-A, Kennedy H, Wang X-J. A large-scale circuit mechanism for hierarchical dynamical processing in the primate cortex. Neuron. 2015;88(2):419–31. doi: 10.1016/j.neuron.2015.09.008 26439530 PMC4630024

[53] Vidaurre D, Smith SM, Woolrich MW. Brain network dynamics are hierarchically organized in time. Proc Natl Acad Sci U S A. 2017;114(48):12827–32. doi: 10.1073/pnas.1705120114 29087305 PMC5715736

[54] Bloem IM, Ling S. Normalization governs attentional modulation within human visual cortex. Nat Commun. 2019;10(1):5660. doi: 10.1038/s41467-019-13597-1 31827078 PMC6906520

[55] Herrmann K, Montaser-Kouhsari L, Carrasco M, Heeger DJ. Nat Neurosci. 2010;:1554–61. 21057509 10.1038/nn.2669 PMC3058765

[56] Herrmann K, Heeger DJ, Carrasco M. Feature-based attention enhances performance by increasing response gain. Vision Res. 2012;74:10–20. doi: 10.1016/j.visres.2012.04.016 22580017 PMC3427403

[57] Doostani N, Hossein-Zadeh G-A, Vaziri-Pashkam M. The normalization model predicts responses in the human visual cortex during object-based attention. Elife. 2023;12:e75726. doi: 10.7554/eLife.75726 37163571 PMC10229119

[58] De Valois RL, Cottaris NP, Mahon LE, Elfar SD, Wilson JA. Spatial and temporal receptive fields of geniculate and cortical cells and directional selectivity. Vision Res. 2000;40(27):3685–702. doi: 10.1016/s0042-6989(00)00210-8 11090662

[59] Sun Y, Nern A, Franconville R, Dana H, Schreiter ER, Looger LL, et al. Neural signatures of dynamic stimulus selection in *Drosophila*. Nat Neurosci. 2017;20(8):1104–13. doi: 10.1038/nn.4581 28604683

[60] Ringach D, Shapley R. Reverse correlation in neurophysiology. Cogn Sci. 2004;28(2):147–66. doi: 10.1207/s15516709cog2802_2

